# Field Performance Test of an Air-Cleaner with Photocatalysis-Plasma Synergistic Reactors for Practical and Long-Term Use

**DOI:** 10.3390/molecules191117424

**Published:** 2014-10-29

**Authors:** Tsuyoshi Ochiai, Erina Ichihashi, Naoki Nishida, Tadashi Machida, Yoshitsugu Uchida, Yuji Hayashi, Yuko Morito, Akira Fujishima

**Affiliations:** 1 Kanagawa Academy of Science and Technology, KSP East 407, 3-2-1 Sakado, Takatsu-ku, Kawasaki, Kanagawa 213-0012, Japan; E-Mail: fujishima_akira@admin.tus.ac.jp; 2 Photocatalysis International Research Center, Tokyo University of Science, 2641 Yamazaki, Noda, Chiba 278-8510, Japan; E-Mail: y.morito@u-vix.com; 3 I’m PACT World Co. Ltd., Domicile 301, 1-3-1 Sugeinadazutsumi, Tama-ku, Kawasaki, Kanagawa 214-0003, Japan; E-Mails: ichihashi@impact-world.jp (E.I.); hayashi@impact-world.jp (Y.H.); 4 Tanashin Denki Co. Ltd., 8-19-20 Fukasawa, Setagaya-ku, Tokyo 158-0081, Japan; E-Mails: nishidan@tanashin.co.jp (N.N.); machida@tanashin.co.jp (T.M.); uchiday@tanashin.co.jp (Y.U.); 5 U-VIX Corporation, 2-14-8 Midorigaoka, Meguro-ku, Tokyo 152-0034, Japan

**Keywords:** photocatalysis, plasma treatment, synergy, air-cleaner, field test, total suspended particulates, total volatile organic compounds, long-term usability

## Abstract

A practical and long-term usable air-cleaner based on the synergy of photocatalysis and plasma treatments has been developed. A field test of the air-cleaner was carried out in an office smoking room. The results were compared to previously reported laboratory test results. Even after a treatment of 12,000 cigarettes-worth of tobacco smoke, the air-cleaner maintained high-level air-purification activity (98.9% ± 0.1% and 88% ± 1% removal of the total suspended particulate (TSP) and total volatile organic compound (TVOC) concentrations, respectively) at single-pass conditions. Although the removal ratio of TSP concentrations was 98.6% ± 0.2%, the ratio of TVOC concentrations was 43.8% after a treatment of 21,900 cigarettes-worth of tobacco smoke in the field test. These results indicate the importance of suitable maintenance of the reactors in the air-cleaner during field use.

## 1. Introduction

**Figure 1:**
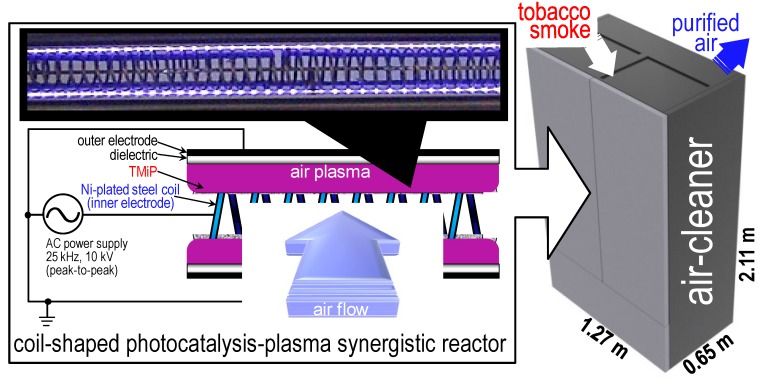
Image and schematic illustration of the coil-shaped PACT-TMiP synergistic reactor (**left**) and the air-cleaner (**right**). Reproduced from Ochiai *et al.* [4], published byScientific Research Publishing Inc., 2014.

**Figure 2:**
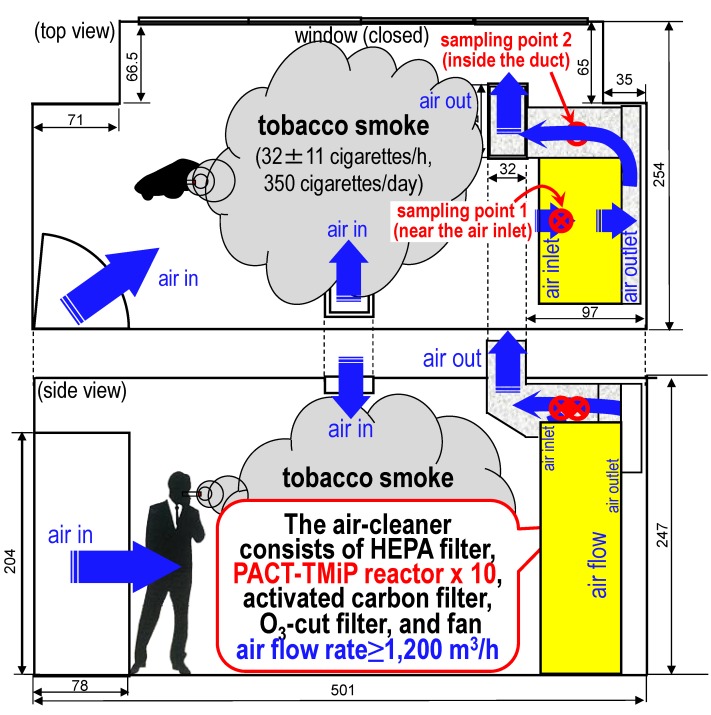
Schematic illustration of the test method in the smoking room for evaluating the air-purification ability of the air-cleaner (unit: cm). Sampling point (1) is near the air inlet of the air-cleaner, and (2) is inside the duct. Detailed experimental procedures are included in Section 3.

Photocatalysis-plasma synergistic reactors have been recently proposed for use in air-cleaners [1,2,3,4]. The synergistic effects of photocatalysis and plasma excitation achieve significant oxidative decomposition of gaseous compounds in laboratory tests. The coil-shaped reactor (Figure 1), using plasma-assisted catalytic technology (PACT) [5] and a TiO_2_ impregnated Ti-mesh filter (TMiP^TM^) [6], shows long-term capability of removing tobacco smoke compounds. High-level air-purification activity was maintained in the air-cleaner with the coil-shaped reactor (≥98, 98.9% ± 0.1%, and 88% ± 1% removal of the odour concentration, total suspended particulate (TSP), and total volatile organic compound (TVOC) concentrations, respectively) even after the treatment of 12,000 cigarettes-worth of tobacco smoke, which is the equivalent of using the air-cleaner in the smoking room for 6 months [4]. In this study, a field test of the air-cleaner using the coil-shaped reactor was carried out for 84 days in a functioning smoking room (Figure 2) of a typical office building. The air-purification ability and the long-term usability of the air-cleaner in the smoking room were discussed by comparison with previously reported laboratory test results.

## 2. Results and Discussion

### 2.1. Evaluations of the Photocatalysis-Plasma Synergistic Air-Cleaner

**Figure 3:**
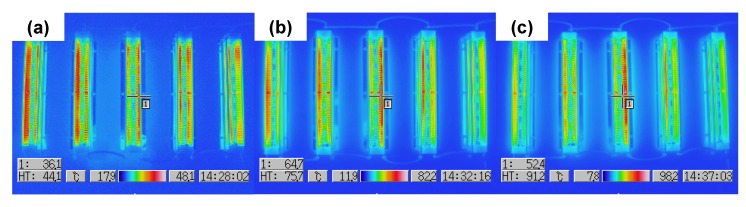
Temperature distribution of the coil-shaped reactorsin the air-cleaner (**a**) 1; (**b**) 5; and (**c**) 10 min after the device is switched on without air flow.

Figure 3 shows the temperature distributions of the coil-shaped reactors in the air-cleaner 1, 5, and 10 min after the device is switched on without air flow. The temperature of the reactors reached an almost steady state at around 100 °C for 10 min (Figure 3c). This temperature is lower than the anatase-to-rutile transformation temperature of TiO_2_ [7]. Therefore, the TiO_2_ photocatalyst on the TMiP surface cannot be affected by the air-plasma. Conversely, ozone and NO_x_ concentrations near the reactors in the air-purifier were 8–9 and 0.7–0.8 ppm, respectively under a 1200 m^3^/h flow rate. These species can accelerate the decomposition of TVOCs [1]. However, ozone and NO_x_ concentrations at the air-outlet of the air-cleaner were below the detection limit. Thus, the ozone-cut and activated carbon filters shown in Figure 2 can reduce excess ozone and NO_x_.

### 2.2. Usage of the Smoking Room

**Figure 4:**
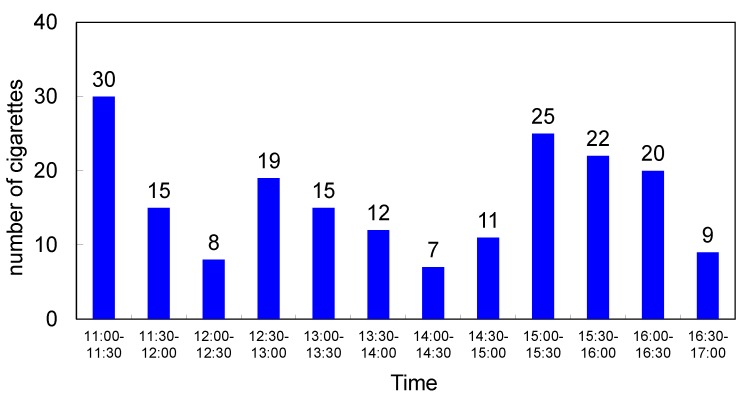
Summary of the number of cigarettes burned every 30 min during the working day (11:00–17:00, 1 May 2014).

The field test was carried out in a smoking room with a volume of approximately 31 m^3^ (5.0 m × 2.5 m × 2.5 m), which was used by several smokers (Figure 2). The number of cigarettes burned every 30 min, from 11:00 to 17:00 during the working day, was counted and the results summarised in Figure 4. On average, 32 ± 11 and 350 cigarettes are burned on an hourly and daily basis, respectively. Generally, an estimated 12,000 cigarettes are burned in the smoking room every six months [4]. Therefore, the number of cigarettes burned in the smoking room in this study is around six times higher than in an average smoking room.

### 2.3. TSP Removal

**Figure 5:**
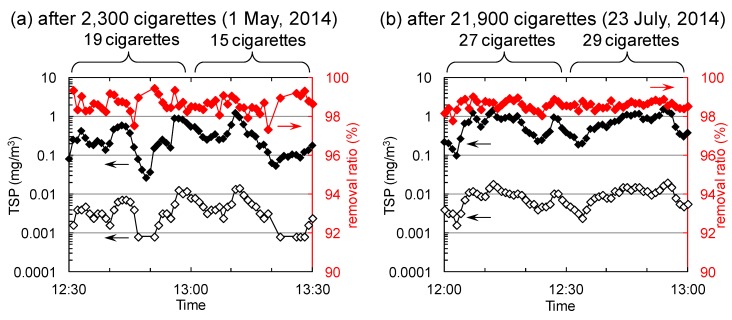
TSP concentrations at sampling points 1 (black filled diamonds, Figure 2) and 2 (black open diamonds, Figure 2) and the removal ratios of TSPs (red filled diamonds) after the treatment of (**a**) 2300 and (**b**) 21,900 cigarettes-worth of tobacco smoke.

Figure 5a shows TSP concentrations at sampling points 1 (near the air inlet of the air-cleaner, Figure 2) and 2 (inside the duct, Figure 2) after the treatment of 2300 cigarettes-worth of tobacco smoke. TSP concentrations at sampling point 1 fluctuated from 0.026 to 1.24 mg/m^3^ with changes in the number of cigarettes burned. The TSP concentrations at sampling point 2 fluctuated from below the detection limit (0.0008 mg/m^3^) to 0.014 mg/m^3^. Thus, the average removal ratio of TSPs was 98.7% ± 0.4%. Moreover, the removal ratio maintained high-levels (98.6% ± 0.2%) after the treatment of 21,900 cigarettes (Figure 5b). These values indicate that the air-cleaner is able to remove TSPs efficiently, in agreement with previously reported air-cleaner trends observed in laboratory test [4].

### 2.4. TVOC Removal

**Figure 6:**
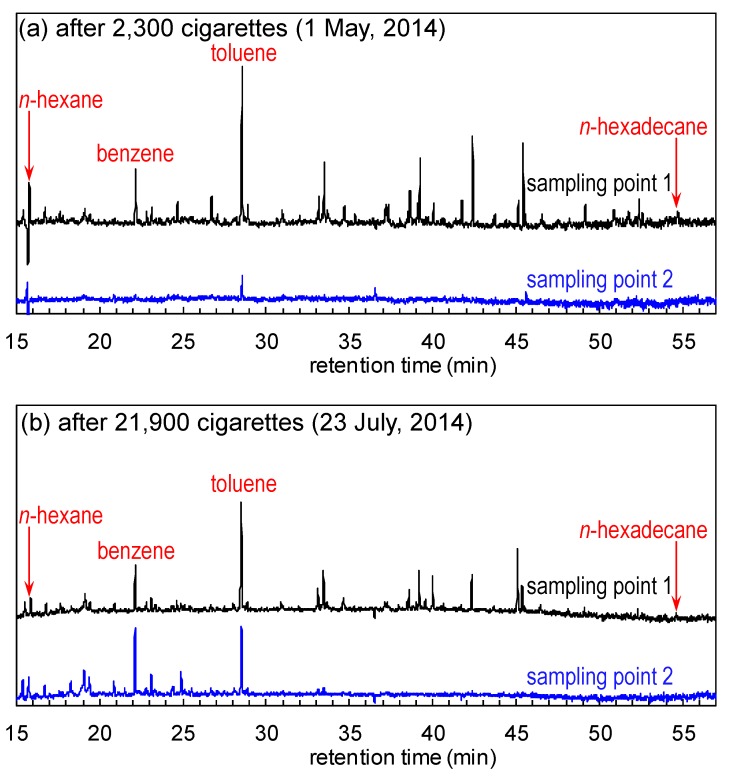
Normalised GC-MS chromatograms of the air samples from the Figure 2 sampling points 1 (black) and 2 (blue) after the treatment of (**a**) 2300 and (**b**) 21,900 cigarettes-worth of tobacco smoke.

Figure 6a shows the normalised GC-MS chromatograms of the air samples at sampling points 1 and 2 after the treatment of 2300 cigarettes-worth of tobacco smoke. Many distinctive VOC peaks were observed in the chromatogram of the air sample at sampling point 1, which had almost disappeared at point 2. TVOC concentrations were calculated for all peaks between *n*-hexane (16.1 min) and *n*-hexadecane (54.7 min); they were then calibrated and converted to toluene peak (28.5 min) equivalents. In Figure 6a, the TVOC concentrations at sampling points 1 and 2 were 128.1 and 3.4 μg/m^3^, respectively (corresponding to a 97.3% removal ratio of TVOCs). However, several peaks remained or were amplified in the chromatogram of the sample at point 2 following the treatment of 21,900 cigarettes-worth of tobacco smoke (Figure 6b), especially between the *n*-hexane (16.1 min) and toluene (28.5 min) peaks. The TVOC removal ratio calculated from Figure 6b dramatically decreased to 43.8%, while the ratio for TSPs did not decrease (Figure 5). These data indicate that the TVOC removal efficiency of the air-cleaner under the present conditions is more easily affected than TSP removal efficiency by catalyst poisoning and the adsorption/desorption of VOCs on the filters during long-term use [8,9,10,11].

### 2.5. Comparison of the Field and Laboratory Tests; The Problems and Future Directions

**Figure 7:**
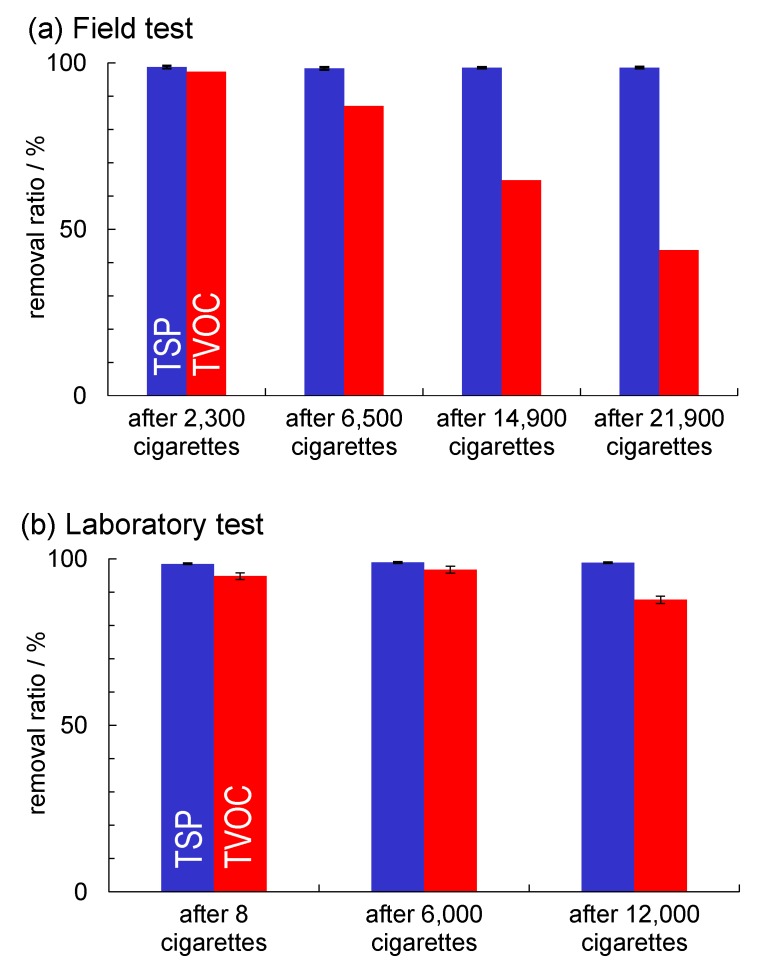
Removal ratios of TSPs (blue) and TVOCs (red) in tobacco smoke by the air-cleaner (**a**) in the field and (**b**) in laboratory tests. Panel (b) is reproduced from Ochiai *et al.*, [4], published byScientific Research Publishing Inc., 2014.

The removal ratios of the TSPs and TVOCs from tobacco smoke by the air-cleaner in the field laboratory tests are summarised in Figure 7. In both the field and the laboratory tests, TSP removal ratios continued at high-levels (around 98.5%) throughout the experimental period. However, the TVOC removal ratios decreased with increases in the number of cigarettes. Intriguingly, the TVOC removal ratios in the field test decreased sooner than in the laboratory test. In this case, TSPs were removed by the HEPA filter and electrostatic precipitation in the plasma treatment [12,13]. However, as mentioned in Section 2.4, TVOC removal was easily affected by catalyst poisoning and adsorption/desorption of VOCs on the filters during long-term field use. To improve long-term usability, suitable maintenance methods must be developed such as plasma ashing of the reactor surfaces.

**Figure 8:**
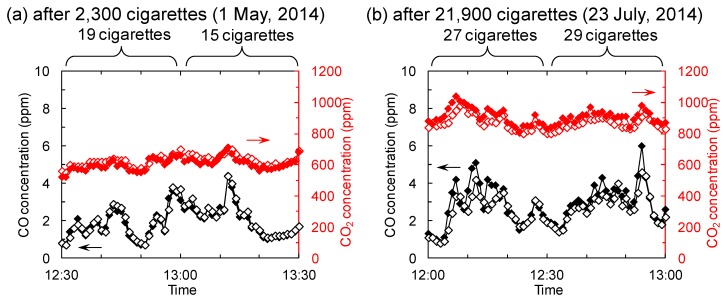
CO (black) and CO_2_ (red) concentrations from the Figure 2 sampling points 1 (filled diamonds) and 2 (open diamonds) after the treatment of (**a**) 2300 and (**b**) 21,900 cigarettes-worth of tobacco smoke.

Another issue that must be considered is CO removal. Figure 8 shows CO and CO_2_ concentrations at sampling points 1 and 2. The concentrations fluctuated with changes in the number of burning cigarettes, as was observed with the TSP concentrations (Figure 5). However, there are no clear differences between the concentrations at points 1 and 2 after the treatment of 2300 and 21,900 cigarettes-worth of tobacco smoke. These data indicate that the present experimental conditions of the photocatalysis-plasma synergistic reactor were not adequate for CO removal, despite the success with the TSP and TVOC removal. Several studies have investigated the synergistic effects of catalysis-plasma or photocatalysis-plasma systems. They found that synergism existed extensively but only under specific conditions [14,15,16,17,18]. Hence, a number of factors have been suggested that can affect efficiency such as catalyst loading level, input power, temperature, adsorption process, *etc*. These factors can be easily influenced by the smoking room usage in this study. This may be the biggest drawback for developing a versatile and effective air-cleaner with a photocatalysis-plasma synergistic reactor. Currently, the oxidation of CO to CO_2_ in the presence of noble metals is being studied for the development of effective catalytic converters and fuel cells [19,20,21]. In this study, the causes of the decrease in TVOC removal efficiency and the poor CO removal efficiency are still unclear. However, more suitable experimental conditions and combinations of catalysis, photocatalysis, and the plasma treatment for effective TVOC and CO removal in the field are being tested.

## 3. Experimental Section

### 3.1. Fabrication and Evaluation of the Photocatalysis-Plasma Synergistic Reactor and the Air-Cleaner

The image and schematic of the coil-shape PACT-TMiP synergistic reactor and air-cleaner are shown in Figure 1. The basic design and fabrication methods of the reactor and the air-cleaner have been previously described [4]. In this study, a voltage of 10 kV (peak-to-peak), a frequency of 25 kHz, and a power of 45 W were used. Air can be drawn through the gaps of the reactor while maintaining high surface contact with TMiP and air-plasma. A high efficiency particulate air (HEPA) filter, ten coil-shape reactors, two ozone-cut filters, an activated carbon filter, and a fan were arranged inside the air-cleaner. When the fan is turned on, air flow is generated inside the casing from the air inlet towards the air outlet, passing through the filters and the PACT-TMiP reactor. Temperature distributions of the coil-shaped reactors in the air-cleaner were measured by thermography using a Handy Thermo TVS-200EX (Nippon Avionics Co., Ltd., Tokyo, Japan). Ozone and NO_x_ concentrations were monitored using a Model 106-L ozone monitor (2B Technologies, Boulder, CO, USA) and MODEL42 I NO_x_ Analyser (Thermo Fisher Scientific, Waltham, MA, USA), respectively.

### 3.2. The Evaluation Method of the Air-Purification Activity by the Air-Cleaner in the Smoking Room

The schematic of the test method for evaluating the air-purification activity of the air-cleaner is shown in Figure 2. Air flow is generated inside the smoking room from the door and air inlet to the duct, passing through the air-cleaner. Under these conditions, the smoking room was filled with tobacco smoke from the sequential burning of cigarettes by the smokers (32 ± 11 cigarettes/h, 350 cigarettes/d). The concentrations of TSPs, TVOCs, carbon monoxide (CO), and carbon dioxide (CO_2_) were measured after the treatments of 2300, 6500, 14,900, and 21,900 cigarettes-worth of tobacco smoke at sampling points 1 (near the air inlet) and 2 (inside the duct) defined in Figure 2. TSP concentrations were monitored using a digital real-time LD-3K2 dust monitor (Sibata Scientific Technology Ltd., Saitama, Japan) every minute for an hour. TVOC concentrations were calculated by qualitative and quantitative analysis using GC-MS analysis. A GC-17A-GCMS-QP5050A combination (Shimadzu, Kyoto, Japan) was used at an ionization voltage of 70 eV and a mass range of 35–200. The system was equipped with a 60 m × 0.25 mm internal diameter × 1.4 μm DB-624 fused silica capillary column (Agilent Technologies, Santa Clara, CA, USA) with split injection (split ratio 11:1). The oven was programmed to start at 35 °C (for 15 min) reaching 240 °C (for 8 min) at a rate of 6 °C/min. Samples were collected by drawing 60 L (0.6 L/min) of air through a charcoal tube, desorbed with 1 mL of carbon disulphide, and analysed by GC-MS. TVOC concentrations were calculated for all compounds eluted between *n*-hexane and *n*-hexadecane, they were then calibrated and converted to toluene equivalents. The removal ratios were calculated using the formula (A_1_ − A_2_)/A_1_, where A_1_ and A_2_ are the amounts at sampling points 1 and 2, respectively. An important point to note is that the tobacco smoke was treated by the air-cleaner once, *i.e*., this was a single-pass system. CO and CO_2_ concentrations were also measured using a COX-3 CO/CO_2_ analyser (Sibata Scientific Technology Ltd., Saitama, Japan) every minute for an hour.

## 4. Conclusions

The photocatalysis-plasma synergistic air-cleaner and its long-term usability in the field were investigated. Compared with previously reported laboratory test results for the air-cleaner, TSP removal ratios remained at high-levels (around 98.5%) throughout the experimental period in both the field and laboratory tests. However, the TVOC removal ratios in the field test decreased three times sooner than in the laboratory test. Additionally, the CO removal ability of the air-cleaner was almost negligible. In conclusion, these results indicate that the photocatalysis-plasma synergistic air-cleaner was effective in the long-term removal of TSPs, given the tobacco smoke conditions in the smoking room investigated. Suitable maintenance methods for the reactor surfaces would improve the long-term TVOC removal ability of the air-cleaner in the field.
